# Revascularization for Chronic Limb-Threatening Ischemia—Synthesizing Inconsistency

**DOI:** 10.1016/j.jscai.2025.102623

**Published:** 2025-04-01

**Authors:** Scott Kinlay

**Affiliations:** a Cardiology Division, Department of Medicine, VA Boston Healthcare System, Boston, Massachusetts; b Cardiovascular Division, Department of Medicine, Massachusetts General Brigham, Boston, Massachusetts; c Harvard Medical School, Boston, Massachusetts

**Keywords:** BASIL-2, BEST-CLI, chronic limb-threatening ischemia, endovascular, open surgical bypass, peripheral artery disease

Arterial revascularization of lower limbs with chronic limb-threatening ischemia (CLTI) is vital to avoid major above-the-ankle amputation. Preventing limb loss preserves quality of life, walking function, and independence. The 2 major forms of revascularization are endovascular therapy and open surgical bypass. Endovascular therapy uses balloons, atherectomy, stents, and other devices. Open surgical bypass uses good quality greater saphenous vein (GSV) or lesser quality grafts such as composite or arm vein, or prosthetic grafts. Each method of revascularization has its proponents.

Outcomes after open surgical revascularization with prosthetic or other vein grafts are clearly worse than with GSV and no better than endovascular revascularization.^1^ The focus in recent years is whether open surgical bypass with GSV is better than endovascular therapy for CLTI. In the past 3 years, 2 randomized trials comparing both methods of revascularization for CLTI should have resolved this conundrum; instead, they reached diametrically opposite conclusions. In the Best Endovascular versus Best Surgical Therapy in Patients with Critical Limb Ischemia (BEST-CLI) trial, major adverse limb events (MALE) or death were lower with open surgical bypass with GSV (of whom 85% actually had GSV).^1^ In the Bypass versus Angioplasty in Severe Ischemia of the Leg-2 (BASIL-2) trial, endovascular therapy had lower rates of death or major amputation compared with open bypass surgery with GSV (of whom 89% had GSV).^2^ How can these 2 major trials be reconciled?

In this issue of *JSCAI*, Wahood et al^3^ report on 30-day outcomes of patients with CLTI treated by vascular surgeons from the National Surgical Quality Improvement Project (NSQIP). This extensive database collects preoperative and intraoperative variables and 30-day outcomes of surgical cases in the United States. Similar to the BEST-CLI trial, Wahood et al^3^ report a higher risk of major amputation and MALE but a lower risk of major cardiovascular events (MACE) over 30 days with endovascular versus open bypass with GSV. There were important differences among the groups in this observational data set. Patients who had open bypass with GSV were younger, had less diabetes mellitus, more functional independence, less hypertension, less bleeding disorders, and more often reported current smoking. Former smoking was not reported, although this is associated with older age and adverse outcomes.^4^ Was open surgical bypass with GSV better, or were vascular surgeons in NSQIP choosing lower-risk patients for this operation and higher-risk patients for endovascular revascularization? Although statistical adjustment can reduce the effects of covariates on outcomes, it cannot adjust for factors that influence the choice of revascularization.

It is tempting to try and discern the differences between BEST-CLI and BASIL-2. Although both studies included patients with infrainguinal disease, the open surgical arms of BEST-CLI and BASIL-2 were different, with femoral-popliteal bypasses in 22% versus 2% of patients and tibial bypasses in 51% versus 97% of patients. The endovascular arms were more similar with superficial femoral artery interventions in 67% versus 46%, popliteal interventions in 53% versus 71%, and tibial interventions in 51% versus 52% of patients. The operators were also different, reflecting transatlantic patterns of who does endovascular therapy for peripheral artery disease. In BEST-CLI, 73% of endovascular procedures were by vascular surgeons, whereas in BASIL-2, 84% were by interventional radiologists. Whether the differences between the trials can be explained by poorer outcomes with tibial artery bypass or who does the endovascular intervention may never be resolved.

**Figure 1:**
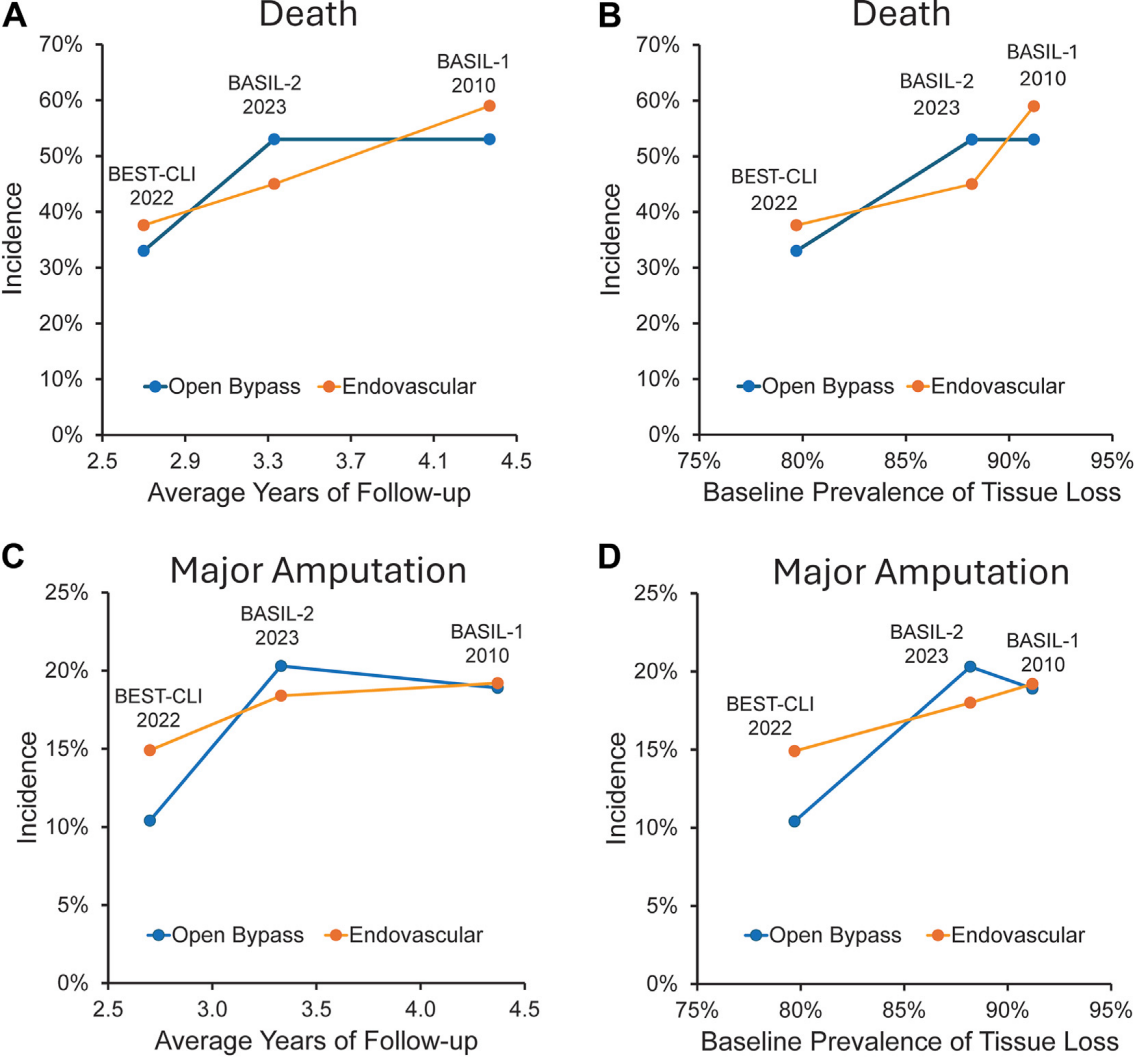
**Outcomes in the BASIL-1, BEST-CLI, and BASIL-2 trials.** Crude rates of death (**A**, **B**) and major amputation (**C**, **D**) by average length of follow-up (**A**, **C**) and the baseline prevalence of tissue loss (**B**, **D**) in BASIL-1^6^^,^^7^ over 4.3 years (published in 2010), BEST-CLI^1^ over 2.7 years (published in 2022), and BASIL-2^2^ over 3.3 years (published in 2023).

Direct comparisons of the outcomes between the trials are difficult. MALE or death was the primary end point for BEST-CLI, and major amputation or death was the primary end point for BASIL-2. Neither trial reported the primary end point of the other trial. Looking at the incidence of death and major amputation, it is clear that outcomes are miserable and have changed little in comparison to BASIL-1,^5^ which was completed more than 2 decades ago (Figure 1).^1^^,^^2^^,^^6^^,^^7^ During follow-up, 30% to 50% of patients died, a similar proportion had a MALE event, 10% to 20% had major amputations, and 40% had a MACE event.^1^^,^^2^^,^^6^^,^^7^ Even a simple analysis shows most of the differences between the trials in the incidence of death and major amputation may be explained by differences in the length of follow-up and the baseline prevalence of tissue loss or gangrene (Figure 1).^1^^,^^2^^,^^6^^,^^7^ The latter is not surprising, as this is probably a marker of more extensive vascular disease and the risk of amputation. Moreover, the absolute differences in these outcomes between open surgery with GSV and endovascular revascularization are relatively small compared with the overall carnage CLTI wreaks on our patients (Figure 1).^1^^,^^2^^,^^6^^,^^7^ Given the opposing results of the trials, arguing about the mode of revascularization or the operator is about as useful as putting cupholders on a bobsled.

Trying to synthesize clear messages from inconsistent trials is difficult. The BEST-CLI and the NSQIP analysis by Wahood et al^3^ indicate that experienced vascular surgeons get their best results treating CLTI with open surgical bypass with GSV. In a similar vein, interventional cardiologists, radiologists, and surgeons who are experienced endovascular proceduralists may have better results with endovascular revascularization as indicated by BASIL-2 and other longer-term cohort studies.^8^^,^^9^

Where does this leave us in the management of CLTI? Factors not addressed by the trials are likely important determinants of clinical outcomes. Given that many outcomes such as death and MACE have little to do with the leg and more to do with the underlying atherosclerotic disease,^8^^,^^10^ specialists in vascular medicine, cardiology, and interventional cardiology are needed to optimize medical management to prevent cardiovascular events. Preserving a limb also depends on managing wounds, and wound specialists and podiatrists are an essential part of the care team to avoid major amputations. For these reasons, centers where these specialties and vascular surgery openly collaborate rather than compete will likely have better outcomes.

Finally, in the light of 2 randomized trials with opposite results, blanket statements that open surgery with GSV or endovascular revascularization are better are oversimplifications and only enflame rivalries to the detriment of our patients. Recognizing the skills of colleagues who may provide a better bypass operation and others who may provide a better endovascular procedure is an altruistic decision that puts the patient first and egos last.
